# Cardiac phase-resolved late gadolinium enhancement imaging

**DOI:** 10.3389/fcvm.2022.917180

**Published:** 2022-09-29

**Authors:** Sebastian Weingärtner, Ömer B. Demirel, Francisco Gama, Iain Pierce, Thomas A. Treibel, Jeanette Schulz-Menger, Mehmet Akçakaya

**Affiliations:** ^1^Department of Imaging Physics, Delft University of Technology, Delft, Netherlands; ^2^Department of Electrical and Computer Engineering, University of Minnesota, Minneapolis, MN, United States; ^3^Center for Magnetic Resonance Research, University of Minnesota, Minneapolis, MN, United States; ^4^Bart's Heart Centre, St. Bartholomew's Hospital, London, United Kingdom; ^5^Institute of Cardiovascular Science, University College London, London, United Kingdom; ^6^Working Group on Cardiovascular Magnetic Resonance Imaging, Experimental and Clinical Research Center, Joint Cooperation of the Max-Delbrück-Centrum and Charite-Medical University Berlin, Berlin, Germany; ^7^Department of Cardiology and Nephrology, HELIOS Klinikum Berlin-Buch and DZHK, Berlin, Germany

**Keywords:** cardiac magnetic resonance (CMR), T1 mapping, LGE imaging, myocardial tissue characterization, magnetic resonance imaging, MRI sequence development

## Abstract

Late gadolinium enhancement (LGE) with cardiac magnetic resonance (CMR) imaging is the clinical reference for assessment of myocardial scar and focal fibrosis. However, current LGE techniques are confined to imaging of a single cardiac phase, which hampers assessment of scar motility and does not allow cross-comparison between multiple phases. In this work, we investigate a three step approach to obtain cardiac phase-resolved LGE images: (1) Acquisition of cardiac phase-resolved imaging data with varying **T**_**1**_ weighting. (2) Generation of semi-quantitative ${\text{T}}_{\text{1}}^{\text{*}}$ maps for each cardiac phase. (3) Synthetization of LGE contrast to obtain functional LGE images. The proposed method is evaluated in phantom imaging, six healthy subjects at 3T and 20 patients at 1.5T. Phantom imaging at 3T demonstrates consistent contrast throughout the cardiac cycle with a coefficient of variation of 2.55 ± 0.42%. *In-vivo* results show reliable LGE contrast with thorough suppression of the myocardial tissue is healthy subjects. The contrast between blood and myocardium showed moderate variation throughout the cardiac cycle in healthy subjects (coefficient of variation 18.2 ± 3.51%). Images were acquired at 40–60 ms and 80 ms temporal resolution, at 3T and 1.5, respectively. Functional LGE images acquired in patients with myocardial scar visualized scar tissue throughout the cardiac cycle, albeit at noticeably lower imaging resolution and noise resilience than the reference technique. The proposed technique bears the promise of integrating the advantages of phase-resolved CMR with LGE imaging, but further improvements in the acquisition quality are warranted for clinical use.

## 1. Introduction

Late gadolinium enhancement (LGE) is the clinical gold standard for assessment of myocardial viability (1, 2) forming an integral part of clinical work up for a wide range of ischemic and non-ischemic cardiomyopathies (3). LGE imaging enables the depiction of myocardial scar by visualizing the retention of a gadolinium based contrast agent. Imaging is performed 10-to-20 min after contrast injection and data is typically acquired during the diastolic quiescence to minimize motion artifacts. In LGE imaging, an inversion recovery sequence is used, where the inversion time is selected to null the signal from the healthy myocardium. This facilitates high contrast of scar tissue as hyper-enhancement against a dark background.

In addition to LGE imaging, most routine clinical cardiac MRI protocols comprise the acquisition of CINE MRI (4, 5). In these scans, data is sampled throughout the cardiac cycle and either prospectively or retrospectively binned into different cardiac phases (6). This allows for reconstruction of phase-resolved images throughout the cardiac cycle. Thus, cine images enable the detailed depiction of cardiac motion, allowing for the quantification of functional parameters and visual assessment of wall motion abnormalities (7).

Thus, LGE and CINE imaging acquisitions differ in terms of their timing and contrast requirements. While CINE imaging is acquired through the cardiac cycle with a steady-state contrast, LGE imaging aims for a particular inversion contrast which is typically specified to coincide with an imaging window in diastole. Nonetheless, acquisition of LGE images at cardiac phases other than end-diastole has proven to be advantageous under certain conditions, as it offers the potential to more clearly depict concealed scar tissue (8, 9). However, due to the use of inversion recovery in traditional LGE imaging, scans are restricted to a single cardiac phase to provide the desired imaging contrast. This inherent limitation prevents joint evaluation of scar and wall motion with traditional LGE sequences. Thus, even though CINE and LGE imaging are the cornerstones of CMR and routinely evaluated alongside each other (3), separate scans are required to comprehensively characterize the myocardium. This not only leads to long scan protocols but also impedes evaluation: Information needs to be fused from separate scans in order to evaluate functional and viability information together. Obtaining phase-resolved LGE images on the other hand has been a long standing aim in cardiac MRI (10, 11) as it may allow for joint evaluation of wall motion abnormalities and viability in a single scan. However, the need for consistent LGE contrast throughout the cardiac cycle has prevented its implementation so far.

Myocardial *T*_1_ mapping was introduced as an alternative for myocardial tissue characterization (12, 13). While sensitivity to ischemic scar remains a subject of ongoing debate (14), native *T*_1_ mapping was demonstrated to provide clinical value in numerous cardiomyopathies (15). Advanced sequence developments have most recently enabled the quantification of myocardial *T*_1_ throughout the cardiac cycle (16–19). Multiple methods have been proposed, including Temporally resolved parametric assessment of Z-magnetization recovery (TOPAZ) (16), phase-resolved cardiac magnetic resonance fingerprinting (MRF) (17, 19), cardiac magnetic resonance multi-tasking (20), and multi-contrast CINE MRI (18). Furthermore, utility of quantitative or semi-quantitative *T*_1_ relaxation information to synthesize LGE imaging contrast has also been explored, showing that synthetic LGE can circumvent the sensitivity to a predefined inversion time that may lead to residual myocardial signal and hamper identification of scar (21–24).

The aim of this study is to integrate these recent developments and enable cardiac phase-resolved LGE imaging with consistent contrast throughout the cardiac cycle. Three steps are proposed to provide phase-resolved viability information in a single scan: First, multiple images with different *T*_1_ weighting are acquired for each cardiac phase, extending on our previously developed TOPAZ technique. Second, semi-quantitative phase-resolved *T*_1_ maps are obtained from this multi-contrast CINE data. Finally, images with the desired LGE imaging contrast are retrospectively synthesized for each cardiac phase, in such a way that the scar is depicted as hyper-enhancement against a dark background of healthy myocardium. Phantom measurements are performed to validate the technique, and image quality is tested in several healthy volunteers and patients at 3T and 1.5T, respectively.

## 2. Materials and methods

### 2.1. Sequence

**Figure 1:**
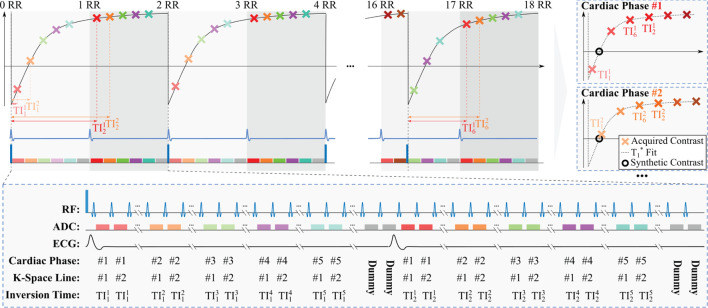
Sequence diagram of the proposed functional LGE sequence. Prior to the inversion pulses shown, magnetization is driven to pulsed steady-state *via* repeated FLASH pulses. Subsequently, an adiabatic pulse is performed to invert the magnetization. Then, contiguous FLASH imaging pulses are played to read out the magnetization during its re-recovery to the pulsed-steady state. The acquisition is segmented, thus, multiple inversion pulses are required to fill the k-space for each cardiac phase. In order to ensure a dense sampling of the inversion recovery curve for each cardiac phase, the Look-Locker experiment is then repeated multiple times with varying inversion pulse offset with respect to the R-wave. As the recovery to the pulsed-steady state spans across two RR-intervals, for each cardiac phase *i* two inversion times $TI_{j}^{i}$ are acquired per inversion pulse offset. Thus, a total of 1 ≤ *j* ≤ 6 inversion times, are acquired per cardiac phase for three inversion pulse offsets. Due to the limited number of inversion pulse offsets, a different sampling of the inversion recovery curve is realized for each cardiac phase, as indicated in the top right panels. Following the acquisition, a semi-quantitative inversion recovery model is fit on these multiple inversion points per cardiac phase to synthesize LGE contrast with suppression of the healthy myocardial tissue.

Figure 1 depicts the sequence diagram of the proposed acquisition scheme, which builds on our recently developed TOPAZ technique (16). In the proposed sequence, the following steps are implemented: (1) The magnetization is driven to pulsed steady-state with continuous FLASH acquisitions. (2) Magnetization inversion is performed. (3) FLASH images are acquired continuously throughout the inversion recovery until pulsed steady-state is re-reached. The magnetization inversion and the imaging readout are prospectively triggered to the R-wave of the ECG to obtain Look-Locker experiments. Low imaging flip angles are used to ensure that the recovery to the steady-state spans two heart beats. In turn, this leads to two points on the inversion curve, separated by an R-R interval, for each inversion pulse. The same Look-Locker experiment is then repeated multiple times, to fill the acquisition k-spaces of all cardiac phases. To ensure an uninterrupted pulsed recovery curve, dummy pulses with no associated imaging readout are performed after the acquisition window. The dummy pulses are played until the R-wave is detected, to ensure there is no gap in pulses before the acquisition window. In the presence of R-R variability, this leads to a variable number of dummy pulses. This way no deviation from the pulsed recovery model occurs and the magnetization is consistently driven to a pulsed steady state, even in the presence of variable R-R durations. The acquisition window, i.e., the time window during an R-R interval in which the prospective data acquisition is performed, is specified to a fixed duration when planning the sequence. Typically the acquisition window duration was chosen around 90% of the R-R interval. (4) The overall experiment is further repeated multiple times while varying the position of the inversion pulse relative to the R-wave. Changing this inversion pulse offset, i.e., the time period between the detection of the R-wave and the application of the inversion pulse, leads to a different time between any given cardiac phase and the preceding inversion pulse. Thus, it enables the acquisition of multiple points on the inversion recovery curve. In the proposed sequence three inversion pulse offsets are acquired. Sampling the inversion recovery curve for each cardiac phase, in turn, allows for semi-quantitative assessment of the longitudinal recovery time and generating synthetic LGE images, as explained below.

For accurate *T*_1_ mapping in the TOPAZ sequence, a three-parameter fit model had to be used to correct for $B_{1}^{+}$-dependent attenuation during the Look-Locker readout. However, in the proposed functional LGE imaging technique, absolute quantification of *T*_1_ or an accurate estimation of $B_{1}^{+}$ are not required, to achieve qualitative LGE contrast after synthetization. Thus, a two-parameter fit model can be employed under the assumption of complete magnetization inversion:


(1) $$\begin{array}{l} S\left(T_{I}\right)=A\left(1-2e^{-\frac{T_{I}}{T_{1}^{*}}}\right), \end{array}$$

where *S*(*T*_*I*_) is the voxel intensity at inversion time *T*_*I*_, and *A* and $T_{1}^{*}$ are model fit parameters, with the latter denoting the apparent *T*_1_ time. Fitting has been performed in a custom tool implemented in C using a Levenberg-Marquardt algorithm from the levmar toolbox for non-linear least-squares fits (25).

Following the fitting procedure, the synthetic LGE image is then generated by applying the following equation voxel-by-voxel:


(2) $$\begin{array}{l} S_{syn}\left(T_{syn}\right)=A\left(1-2e^{-\frac{T_{syn}}{T_{1}^{*}}}\right), \end{array}$$

where *S*_*syn*_ is the synthesized signal, and *A* and $T_{1}^{*}$ are the parameters as obtained from the voxel-wise model fit in Equation (1). *T*_*syn*_ is the synthetic inversion time that is retrospectively chosen to null the signal of the healthy myocardium. A single virtual inversion time is used for all cardiac phases in order to provide consistent LGE contrast and enable cross comparison among the phases.

### 2.2. Simulations

Numerical simulations have been performed to assess the noise resilience at different cardiac phases acquired with the proposed method. Noisy Bloch simulations were performed with a simulated heart rate of 60 bpm. The post-contrast myocardial *T*_1_ time was simulated between 350 and 650 ms. The remaining sequence parameters were chosen to match the phantom and *in vivo* experiments at 3T (Table 1). The noise level was chosen to simulate a baseline SNR of 20 and *N* = 1,000 repetitions were performed. The coefficient of variability was assessed as the standard deviation of the obtained, apparent $T_{1}^{*}$ over its mean.

**Table 1:** Sequence parameters of the proposed functional LGE sequence at 1.5T and 3T.

	**Functional LGE**		**Conventional LGE**	
	**1.5T**	**3T**	**1.5T**	**3T**
Sequence type	FLASH	FLASH	FLASH	bSSFP
TE (ms)	3.2	2.6	4.9	1.1
TR (ms)	6.7	5.0	10.0	2.6–2.8
Flip angle	6°	3°	30°	50°
GRAPPA	2	2	1	2
Partial fourier	6/8	6/8	1	1
Averages	1	1	1	8
In-plane resolution (mm^2^)	2.1 × 2.1	1.9 × 1.9	2.1 × 2.1	1.6 × 1.6
Slice thickness (mm)	10.0	10.0	10	8.0
Temporal resolution (ms)	80	40–60	N/A	N/A
Number of inversion times	6	6	1	1
Field of view (mm^2^)	225 × 300	225 × 300	300 × 300	270 × 360
Number of heart beats	18	18	12	16
Breath-hold duration (s)	15–18	17–19	10–12	14–16
Time between inversion pulses (heart beats)	2	2	2 (PSIR)	2 (PSIR)

N/A indicates not applicable.

### 2.3. Phantom experiments

Phantom experiments were conducted on a 3T Siemens Magnetom Prisma (Siemens Healthcare, Erlangen, Germany) scanner. Imaging was performed with a 30-channel receiver array. The imaging parameters for the functional LGE sequence are listed in Table 1. As previously proposed for TOPAZ (16), flip-angle and TR were chosen using numerical optimizations to obtain an ideal trade-off between signal strength and relaxation rate of the pulsed recovery curve. Two sets of phantom experiments were performed, to study the CNR across the cardiac cycle, and to investigate the effect of different heart rates, respectively.

For the first set of phantom experiments two spheres filled with Gadolinium-doped agarose gel were used. The spheres were constructed to be approximately representative of post-contrast *T*_1_ times in the blood pool and the healthy myocardium [*T*_1_ = 489 ms, 910 ms, respectively, (26)]. The synthetic inversion time was set to *T*_*syn*_ = 567 ms in order to null the signal in the sphere representative of the myocardium. Imaging was performed with a simulated ECG at 60 bpm. Ten repetitions of the functional LGE sequence were acquired in the phantom setting. The contrast-to-noise ratio (CNR) was assessed between manually drawn ROIs in the two spheres. Due to the non-linear processing, noise was defined as pixel wise variability across the ten repetitions. The contrast homogeneity was quantified by analyzing the coefficient of variance (CoV) of the CNR throughout the simulated cardiac cycle.

In the second set of experiments, a bottle phantom was imaged at various simulated heart rates. *T*_*syn*_ was chosen to null the compartment with the longer *T*_1_ time. Heart rates between 50 and 90 bpm were simulated and five repetitions were acquired for each heart rate. The signal in the other phantom compartment was compared across the different heart rates.

### 2.4. *In-vivo* experiments

The imaging protocols were approved by the respective local institutional review boards. Written informed consent was obtained from each subject prior to examination.

Three *in vivo* cohorts have been scanned in this study. First, six healthy subjects (4 male, 2 female, 40 ± 19 years old) have been scanned at 3T (Magnetom Prisma, Siemens Healthineers, Erlangen, Germany). Second, 20 patients (13 male, 7 female, 50 ± 16 years old) were imaged on a 1.5T scanner (Avanto Fit, Siemens Healthineers, Erlangen, Germany). In this cohort patients with suspected or confirmed coronary artery disease who were scheduled to be scanned on one of the three selected scan days have been included. Finally, four patients (1 male, 4 female, 66 ± 7 years old) have been scanned at 3T (Magnetom Prisma, Siemens Healthineers, Erlangen, Germany). Two of those patients have been referred to CMR for potential myocarditis, one for evaluation of coronary artery disease, and one for follow-up of dilated cardiomyopathy.

The differences in scanner hardware, field strength and consequently different required adoption of the imaging parameters. The full set of imaging parameters is provided in Table 1. Additionally, phase-sensitive inversion recovery (27) LGE imaging was performed as reference. The corresponding sequence parameters can be found in Table 1. All LGE imaging was performed 10–20 min after injection of 0.2 mmol/kg gadobutrol (Gadovist, Bayer, Leverkusen, Germany) or 0.1 mmol/kg gadoterate meglumine (Dotarem, Guerbet, Villepinte, France) contrast agent.

Contrast was quantitatively analyzed in all healthy subjects. ROIs were manually drawn in the septum and the left-ventricular blood-pool. Apparent *in-vivo* CNR was defined as follows:


(3) $$\begin{array}{l} \text{aCNR}=\frac{\left|\mu_{Myo}-\mu_{Blood}\right|}{\sqrt{\left(\sigma_{Myo}^{2}+\sigma_{Blood}^{2}\right)/2}}, \end{array}$$

where μ_*Myo*_, μ_*Blood*_ describe the average signal in the ROI drawn in the myocardium and the blood pool, respectively. σ_*Myo*_ and σ_*Blood*_ describe the spatial standard deviation across the ROIs.

In healthy subjects ROIs were drawn for all cardiac phases individually. As the temporal resolution was fixed, a different number of acquisition cardiac phases is acquired for different heart rates. In order to compare the aCNR for subjects with different heart rates, the acquired number of cardiac phases (10–18) was interpolated to 20 reconstruction phases, using linear interpolation. The CoV of the aCNR was assessed throughout the cardiac cycle as a measure of contrast homogeneity.

In the patient cohorts, ROIs were drawn for one diastolic and one systolic phase. The aCNR was then quantitatively compared among the cohorts for both phases. ANOVA was performed to find statistical differences among the aCNR of the groups, and *p* < 0.05 was considered significant.

## 3. Results

### 3.1. Simulations

The results of the numerical simulations are displayed in Supplementary Figure 1. A marked difference in noise resilience is observed across the cardiac cycle. Cardiac phases with minimal TIs close to the nulling point of the simulated *T*_1_, suffer from the most noise variability. Additionally, increasing simulated *T*_1_ times, leads to an overall increase in noise susceptibility, as observed in the coefficient of variability.

### 3.2. Phantom experiments

**Figure 2:**
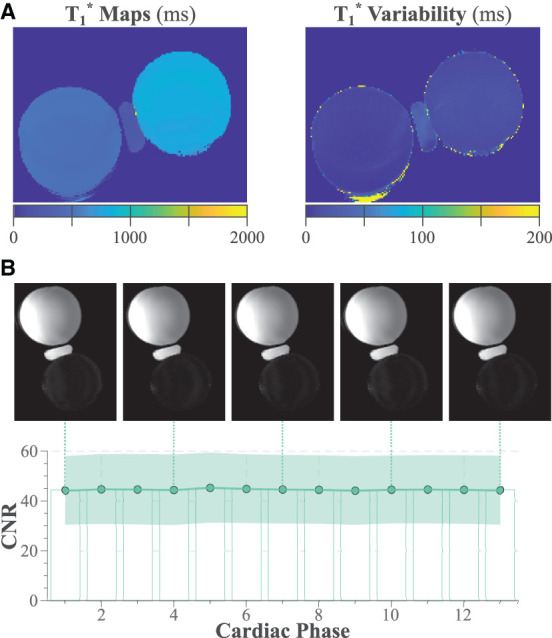
**(A)** $T_{1}^{*}$ acquired in phantom with the proposed functional LGE sequence. The left panel shows the maps averaged across the simulated cardiac cycle, while the right panel displays the variability throughout the cardiac cycle. **(B)** Phantom functional LGE images with the synthetic inversion time chosen to null the myocardial (lower) sphere. The contrast to noise ratio between the two spheres is plotted throughout the simulated cardiac cycle. Shading indicates the spatial variability across the manually drawn ROIs.

Semi-quantitative $T_{1}^{*}$ maps acquired with the proposed functional LGE sequence are displayed in Figure 2A. The maps display the average and variability of the $T_{1}^{*}$ time throughout the cardiac cycle. The CoV of the $T_{1}^{*}$ time was 1.31 ± 0.19 and 1.89 ± 0.23% for the blood and the myocardial sphere, respectively. Figure 2B shows functional LGE images acquired in the phantom throughout the simulated cardiac cycle. The images depict thorough nulling of the myocardial sphere. The CNR is largely constant throughout the simulated cardiac cycle, resulting in a CoV of 2.55 ± 0.42%.

Additionally, only minor variation of the functional LGE signal was observed for different heart rates (Supplementary Figure 2). The CoV of the signal when varying the simulated heart rate from 50 to 90 bpm, was 2.03 ± 0.81%, with no significant trend (*R*^2^ = 0.0013, *p* = 0.865).

### 3.3. *In-vivo* experiments

**Figure 3:**
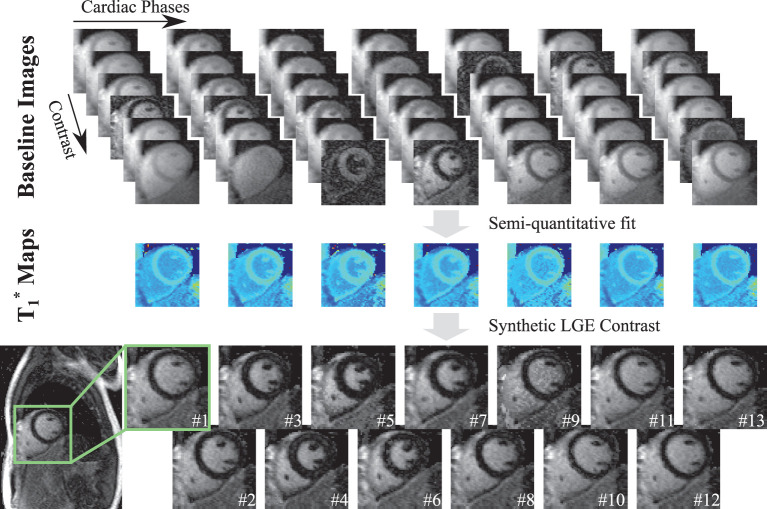
The post-processing pipeline for generating the proposed functional LGE images from the acquired *T*_1_-weighted data. For each cardiac phase, the proposed sequence acquires multiple images with different *T*_1_ weighting, denoted as Baseline Images. A two-parameter fit is performed on these images to obtain semi-quantitative $T_{1}^{*}$ maps for each cardiac phase. Finally, a virtual inversion time is retrospectively chosen to synthesize functional LGE images for all cardiac phases.

Figure 3 depicts an example of the processing pipeline used to obtain functional LGE images in a healthy subject scanned at 3T and 40 ms temporal resolution. Six baseline images with different *T*_1_ weighting are acquired for each cardiac cycle and used to generate the apparent $T_{1}^{*}$ maps. The $T_{1}^{*}$ maps depict visually high image quality with homogeneous $T_{1}^{*}$ times throughout the myocardium and across cardiac cycles. Accordingly, the resulting synthetic functional LGE images depict thorough suppression of the healthy myocardial signal at each phase of the cardiac cycle. Furthermore, clear delineation with respect to the blood pool is visually apparent.

**Figure 4:**
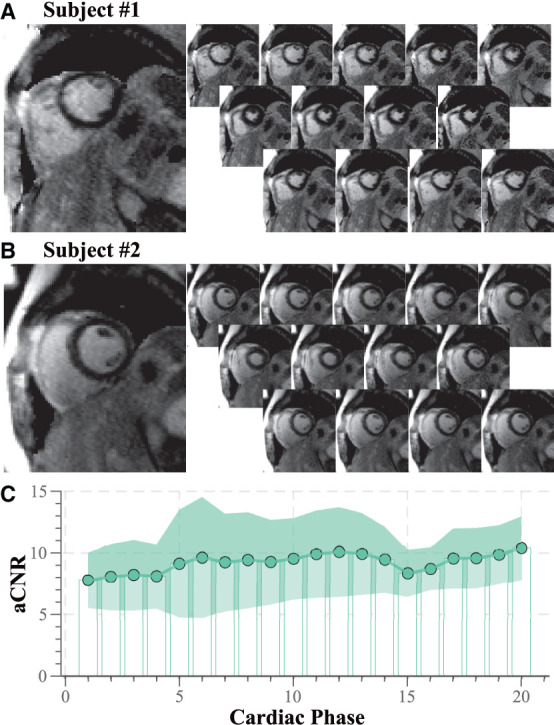
Representative functional LGE images acquired in healthy volunteers at 3T. **(A)** Subject #1 with heart rate 57 bpm, number of acquisition phases 13, **(B)** subject #2 heart rate 57 bpm, number of acquisition phases 13. Thorough suppression of the healthy myocardial tissue with sharp delineation against the blood pools is visible for both subjects. **(C)** Quantitative analysis of aCNR throughout the cardiac cycle across the six healthy volunteers. Moderate variation of the aCNR is obtained across the cardiac phases.

Figure 4 depicts further examples acquired in two healthy subjects at 3T. Consistent nulling of the myocardium is obtained in both healthy subjects. Furthermore, homogeneous contrast with clear depiction of the blood-myocardium contrast is visible throughout the entire cardiac cycle (Supplementary Video 1). Quantitative analysis of the aCNR between the myocardium and the blood pool is depicted in Figure 4C. Moderate variation of the aCNR across the cardiac cycle was observed, amounting to a CoV of 18.2 ± 3.51%.

**Figure 5:**
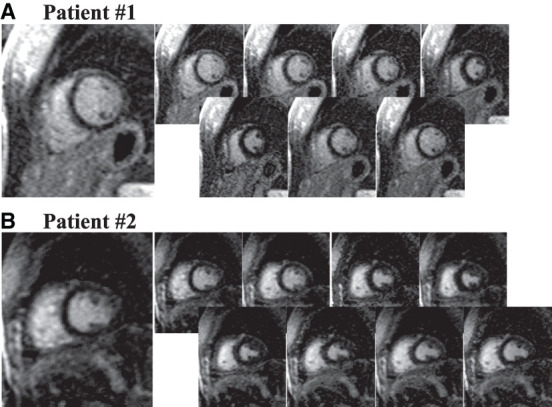
Functional LGE images acquired in LGE negative patients at 1.5T with 80 ms temporal resolution. Compared with 3T visually increased noise is depicted in the 1.5T images due to reduced baseline SNR at this lower field strength, and later acquisition time after contrast injection. While, consistent nulling of the myocardium is achieved and the blood-pools remain clearly depicted. **(A)** Seven cardiac phases were obtained in this patient with a heart rate of 73 bpm. **(B)** Eight cardiac phases were acquired in this subject with 77 bpm heart rate.

Patient images obtained at 1.5T are shown in Figure 5. Both of these patients were LGE negative. Although the myocardial signal is visually suppressed in all cardiac phases, noise variability appears visually higher in some cardiac phases (e.g., phase 3 for both patients). A cinematographic view of all cardiac phases can be found in Supplementary Video 2.

**Figure 6:**
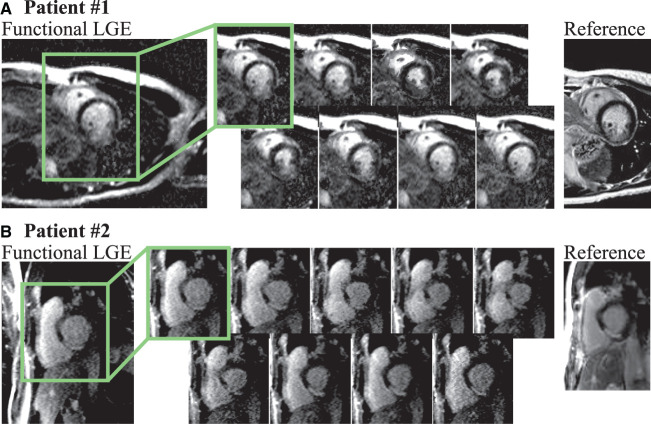
**(A)** Functional LGE images acquired in a 41 year old female patient with known history of CAD and myocarditis in comparison to a reference LGE acquisition (heart rate 80 bpm, acquisition phases 8). The patient displays a large antero-lateral scar that is visible with the proposed technique throughout all cardiac phases. Retrospective choice of the inversion time enables bright scar hyper-enhancement delineated against the remote myocardium. However, compared to the reference scan, decreased imaging resolution hampers depiction of the scar structure. **(B)** Functional LGE images acquired in a patient suffering from CAD and displaying scar in the lateral segment (heart rate 72 bpm, acquisition phases 9). Clear depiction of the scar tissue is achieved in all cardiac phases, albeit at higher noise levels compared with the clinical reference scan.

Figure 6A shows images acquired in a patient with a history of myocarditis and CAD. Antero-lateral scar is visible in the LGE reference scan. The scar is also visually apparent in all phases acquired with the proposed method. Better resolution of the scar structure is achieved with the reference method, and the reference scan appears markedly less noisy. Two cardiac phases (3 and 6) present patch-like artifacts in the blood pools, as a result of the contrast synthetization. Scar motility and contrast throughout the cardiac cycle can be visualized using a cinematographic view of the cardiac phases, as shown in Supplementary Video 3.

Figure 6B depicts a second CAD patient imaged at 1.5T with 80 ms temporal resolution. Both conventional and the proposed functional LGE methods display scar in the lateral segment. Scar tissue can be visually discerned in all cardiac phases of the functional LGE scan. Thus, scar motility is captured and the displacement can be tracked throughout the heart-beat (Supplementary Video 3). However, a noticeably higher level of imaging noise is observed compared with the clinical reference LGE scan. Furthermore, the noise level appears visually exacerbated in later cardiac phases.

**Figure 7:**
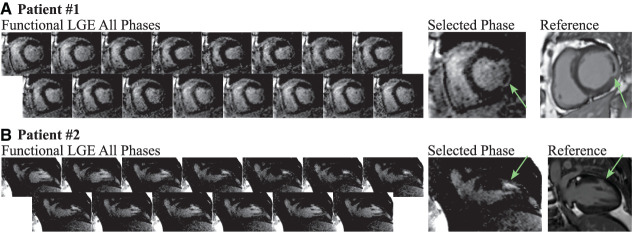
Functional LGE images acquired in two patients at 3T compared to high-resolution reference LGE images. **(A)** Lateral scar (green arrows) is observed in a 73 old woman (heart rate 57 bpm, acquisition phases 16), referred to CMR for evaluation of myocarditis and perfusion defects. The scar tissue is visible in all cardiac phases of the functional LGE scan acquired at 60 ms temporal resolution, albeit variations in the noise levels across the cardiac cycle can be observed. In comparison, the high-resolution reference LGE scan displays much better noise resilience and a finer depiction of the scar structure. **(B)** Two chamber acquisition in a 58 year-old man (heart rate 64 bpm, acquisition phases 13) referred to CMR for evaluation of coronary artery disease. The subendocardial scar (green arrows) can be seen in both the reference and the functional LGE images. Systolic phases of the latter visualize spatially and contrast the separation of the scar tissue from the nearby blood pool.

Figure 7 shows images acquired in two patients 3T with 60 ms temporal resolution. In the first subject (Figure 7A) lateral scar is visually apparent in functional LGE images. However, the scar depiction in the high-resolution reference scan is visually superior and the structure of the scar tissue can be better delineated. Additionally, the functional LGE scan shows elevated noise variability in some cardiac phases (6 and 11). A two chamber view in the second patient (Figure 7A) reveals a subendocardial scar, which is not easily discerned from the blood pool. However, in systolic cardiac phases the scar tissue is well-separated spatially and in terms of contrast, aiding the identification in the functional LGE images.

**Figure 8:**
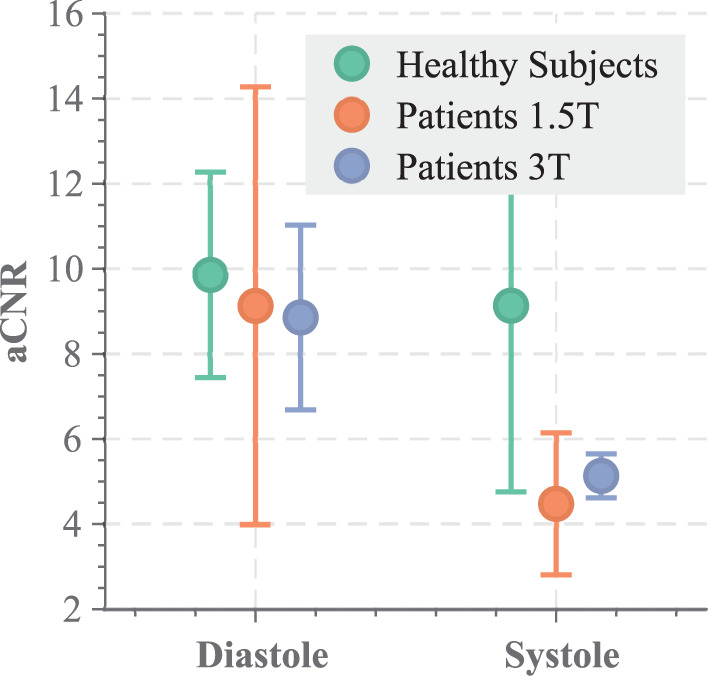
aCNR across the different cohorts, analyzed for a diastolic and systolic phase of the functional LGE scans.

Figure 8 depicts the quantitative comparison of the aCNR across the different cohorts. No significant difference was found for the diastolic phase among the different cohorts (*p* = 0.90). However, the patient cohorts suffer from substantially lower aCNR in the systolic phase, as compared with the healthy subject data (*p* < 0.037), suggesting larger variability in the aCNR throughout the cardiac cycle.

## 4. Discussion

In this study, we proposed a method for augmenting LGE imaging with a functional acquisition. Cardiac phase-resolved LGE images are obtained with consistent contrast throughout the cardiac cycle. In this study, we demonstrated the feasibility of obtaining cardiac phase-resolved images with LGE contrast. Semi-quantitative $T_{1}^{*}$ maps were acquired in a cardiac phase-resolved manner. Subsequently, synthetic image generation was used to obtain the clinical LGE contrast that nulls the healthy myocardium for all cardiac phases. Phantom imaging showed consistent contrast with thorough nulling of the desired signal using the proposed method. Initial *in-vivo* results at 3T demonstrate promising image quality with a temporal resolution of up to 40 ms in healthy subjects. However, initial clinical data at both 1.5T and 3T shows substantially higher noise levels and reduced imaging resolution compared to clinical reference LGE images.

Noise susceptibility and imaging resolution remained a challenge in the clinical cohorts in this study. The drop in image quality in the patient cohort compared to healthy subjects was likely primarily driven by the much later acquisition time after contrast injection, as clinical reference LGE scans were always acquired first. Acquisitions late after contrast injection lead to longer post-contrast *T*_1_ times, due to contrast washout. Our simulation results show that this leads to an overall increase in noise variability with the proposed technique. While imaging contrast was observed to be largely constant, noise susceptibility showed major variations throughout the cardiac cycle. Due to the sequence design, each cardiac phase realizes a different sampling of the inversion recovery curve. Simulation results show that cardiac phases, where the minimal TI is close to the null point of the inversion recovery curve, lead to the highest noise variability. Accordingly, in the patient cohorts, cardiac phases where the first point on the inversion recovery curve is relatively late, appear visually most susceptible to noise. In the present method, the signal polarity is restored prior to fitting using the approach proposed by Messroghli et al. (12). However, it has been previously reported that this can lead to additional noise variability if an inversion time near the zero crossing of the inversion recovery curve is sampled (28). Accordingly, due to inadvertent sampling of the inversion recovery curve, salt-and-pepper-like noise and patchy appearance can be observed in these phases. Using the signal phase to restore the polarity has been proposed as a way to mitigate those sampling-related artifacts. Incorporating this method into the proposed technique bears promise to enable more homogeneous noise resilience across the cardiac cycle and warrants further investigation. While the image quality can still be sufficient to track large areas of hyper-enhancement throughout the cardiac cycle, improvements in the trade-off between spatiotemporal resolution and noise resilience and needed to match reference LGE image quality. An increasing number of methods have emerged that also enable cardiac phase-resolved *T*_1_ mapping, including methods based on cardiac MRF (29) and cardiac multi-tasking (20). These methods attain better baseline map quality by means of regularized or model-based reconstructions. Similarly, regularization approaches have recently been demonstrated to substantially improve noise resilience in TOPAZ (30, 31). Thus, future work is warranted that integrates regularized reconstruction schemes, as proposed for TOPAZ or other phase resolved cardiac *T*_1_ mapping techniques, to enhance noise resilience and spatiotemporal resolution.

Functional LGE imaging has the premise of depicting scar at multiple cardiac phases. Thus, the images allow for cross-comparison of scar signal throughout the cardiac cycle. This may increase diagnostic certainty if ambiguous enhancement is observed, for example in the vicinity of the left-ventricular blood pool or close to fatty tissue. Furthermore, the proposed functional LGE imaging sequence achieved substantially higher temporal resolution than conventional LGE imaging. In standard clinical LGE imaging, diastolic triggering is used, requiring careful manual timing to place the acquisition window (~100–250 ms) into the diastolic quiescence. This temporal resolution might not be ideal when highly mobile structures, such as the papillary muscles, are to be assessed (32). Functional LGE imaging can potentially improve upon these points as a temporal resolution as low as 40–80 ms was achieved. Additionally, since synthetic LGE images are available throughout the cardiac cycle, potentially detrimental imaging artifacts due to incorrect placement of the acquisition window are eliminated. Instead phase-resolved imaging ensures that LGE images are provided at the desired cardiac phase with no need for manual timing. However, additional improvements in image quality may be required to fully replace the reference LGE scan at least at 1.5T.

The most recent recommendations for clinical CMR include CINE MRI of the left ventricle and LGE for almost all ischemic and non-ischemic cardiomyopathies (5). More specifically, when revascularization is considered, for example, only the joint evaluation of cardiac function and viability is considered to offer sufficient information for clinical decision making (3). Together, LGE imaging and CINE MRI, enable the assessment of functional impairment of the scar region and potential links to any wall motion abnormalities. However, as these scans are commonly acquired with two separate sequences, cross evaluation can only be performed subjectively. This hampers the fusion of data and complicates the reading of images. Cardiac phase-resolved LGE images on the other hand, inherently allow for joint assessment of myocardial function and viability in scar and surrounding tissue. Furthermore, previous studies have indicated value of obtaining viability information in parts of the cardiac cycle other than diastole (22, 24, 33). Specifically, it has been shown that this may ease the assessment of scar transmurality (21). Thus, depiction in multiple cardiac phases as achieved with the proposed method bears promise for improved clinical certainty in assessing myocardial scar and warrants further investigation for its prognostic value in the clinical setting. This, however, was beyond the scope of the current work.

Apparent CNR in functional LGE images between myocardium and blood pool was found to be moderately variable throughout the cardiac cycle. This is in-line with previous reports indicating differences in *T*_1_ times at different the cardiac phases (33, 34) and might be explained with differences in the blood-myocardium volume fraction. Due to the non-linear processing of the functional LGE images, noise variability cannot be obtained from background intensity, but was instead defined as the spatial standard deviation within the regions of interest. Thus, variability of physiological and system parameters within the ROI will compromise the reported aCNR values. While the reported aCNR is useful as a metric to compare contrast throughout the cardiac cycle, it should be noted that this hampers comparability of the reported aCNR to literature values of CNR.

The phase-resolved images in this study were acquired with prospective ECG triggering. Prospective and retrospective triggering has a different spectrum of advantages and disadvantages for cardiac phase-resolved imaging. Prospectively triggered image acquisitions have been recognized to be resilient against variabilities in the RR interval, as the duration of the systolic phase commonly remains relatively constant (35). On the other hand, a prospectively defined acquisition window is commonly defined to only cover 80–90% of the cardiac cycle. Thus, the end-diastolic phase may be partially missed, and peak diastolic filling may be underestimated. For those reasons retrospective CINE is commonly preferred for quantification of cardiac function and is most commonly used in clinical practice (5). An increasing number of techniques enabling cardiac phase-resolved quantification of the longitudinal relaxation time have recently been proposed (20). Those methods can be used in combination with retrospective cardiac gating, and may therefore warrant investigation in combination with the proposed approach if the extraction of quantitative cardiac function is desired.

Imaging in this study was performed in a single mid-ventricular slice only. Thus, the functional LGE images obtained in this study have not been evaluated for cardiac volumetry. However, at 3T a temporal resolution up to 40 ms was obtained. This is comparable to the temporal resolution used for 2D CINE MRI in clinical routine (5, 36). At this temporal resolution blurring due to cardiac motion is considered to be minimal (37–39), making CINE MRI suitable for accurate assessment of cardiac function. Hence, functional LGE images may be suitable for quantification of cardiac function, albeit being subject to the specific drawbacks of prospectively triggered CINE with the current sequence implementation, as reported for certain patient groups (40). Alternatively, retrospective ECG gating can be employed in the present sequence design. However, the inversion pulse timing would need to be adapted in real time to ensure the desired semi-quantitative imaging information. Thus, further improvements to tailor the proposed approach for functional volumetry remain a subject of future work.

The proposed method synthesizes LGE contrast based on a range of different inversion contrasts for each cardiac phase. The fusion of multiple data points potentially makes this approach susceptible to residual motion, for example, due to incomplete breath-holds. While this has not been observed to be an issue in the present data set, this issue may be exacerbated in some clinical cohorts. Other previously proposed cardiac phase-resolved *T*_1_ mapping techniques have been proposed as a free-breathing acquisition based on self-gating signals (17, 18, 20). A similar approach could be employed in this sequence to mitigate the need for long breath-holds, avoid susceptibility to incomplete breath-holds, and enable increased sequence durations. Additionally, a range of motion correction techniques has been proposed and successfully applied to quantitative cardiac MRI (41–43). Using these techniques in the proposed method on a phase-by-phase basis to alleviate residual motion, warrants further investigation.

The proposed functional LGE method relies on selection of a synthetic inversion time to achieve LGE imaging contrast. In this study, the remote myocardium that needed to be nulled was visually identified, from which the synthetic inversion time was generated. Recent advances in machine learning have enabled improved tools for automatic identification of such tissues. Specifically for cardiac MRI, numerous methods have been developed to achieve highly accurate segmentation of the cardiac anatomy (44–47). Such methods can be used to automatically delineate the tissue that is to be nulled. Thus, future work will explore the integration of deep-learning based segmentation to enable automatic selection of the synthetic inversion time. This would facilitate LGE scanning without the need for manual timing selection, neither prospectively in the protocol nor retrospectively in the reconstruction.

Black-blood LGE imaging has recently been developed, based on a combination of *T*_1_ and *T*_2_ contrast sensitization (48, 49). The black-blood contrast bears promise for improved depiction of sub-endocardial scar neighboring the blood-pool (50). Combined methods for simultaneous quantification of myocardial *T*_1_ and *T*_2_ times have also been explored (17, 19, 51, 52). By exploiting phase-resolved *T*_1_ and *T*_2_ quantification the proposed method can potentially be extended for the generation of functional black-blood LGE images. This combination remains subject of future work.

The present study and the proposed functional LGE imaging method are subject to several limitations. In this study, only a small number of healthy volunteers was scanned at 3T and no comparison to healthy subjects at 1.5T has been performed, to minimize the use of gadolinium contrast agents in a healthy population. Furthermore, the method was evaluated in a general patient cohort, providing representative examples of feasibility and image quality as encountered in clinical use. However, more specific and larger patient cohorts with LGE need to be assessed in order to evaluate prognostic and diagnostic value of functional LGE as compared to conventional LGE and CINE imaging. In this study only a single mid-ventricular slice was acquired with proposed functional LGE method. Whole heart coverage requires repeated breath-holds, which hampers integration in the existing clinical workflow. Simultaneous-multi slice (SMS) imaging has recently been evaluated for improved spatial coverage in quantitative mapping of the heart (53–55). In functional LGE imaging SMS can facilitate improved coverage in fewer breath-holds for the benefit of easing clinical translation. Future studies will evaluate the use of SMS accelerated scans to obtain whole heart coverage in functional LGE imaging.

## 5. Conclusion

We have demonstrated the feasibility of generating functional LGE imaging with temporal resolution of up to 40 ms. The proposed functional LGE images allowed consistent contrast, nulling the healthy myocardium throughout the cardiac cycle, as well as clear delineation of the myocardium against the blood-pool in healthy subjects at 3T. Initial patient images demonstrate the feasibility of our functional LGE approach to visualize scar motility across multiple cardiac phases. However, spatial resolution and imaging noise were markedly worse in the patient cohort, and further improvements are warranted to match reference LGE image quality. Nonetheless, the proposed technique bears the promise to offer additional insights by enabling a direct depiction of scar tissue motility.

## Data availability statement

The raw data supporting the conclusions of this article will be made available by the authors, without undue reservation.

## Ethics statement

The studies involving human participants were reviewed and approved by the Ethics Committees of the University of Minnesota, HELIOS Klinikum Berlin-Buch, and the Barts Heart Centre. The patients/participants provided their written informed consent to participate in this study.

## Author contributions

SW and MA: study conception and design, analysis and interpretation of results, and draft manuscript preparation. SW: pulse sequence development. SW, ÖD, JS-M, FG, IP, and TT: data collection. All authors reviewed the results and approved the final version of the manuscript.

## Funding

This work was supported in part by the 4TU federation, a NWO Start-up grant STU.019.024, ZonMW Off-Road 04510011910073, NIH R01HL153146, NIH R21EB028369, NIH P41EB027061, and NSF CAREER CCF-1651825. ÖD was partially supported by an AHA Predoctoral Fellowship. TT was funded by a BHF Intermediate Research Fellowship (FS/19/35/34374) and directly and indirectly supported by the UCLH NIHR Biomedical Research Centre and Biomedical Research Unit at UCLH and Barts, respectively.

## Conflict of interest

The authors declare that the research was conducted in the absence of any commercial or financial relationships that could be construed as a potential conflict of interest.

## Publisher's note

All claims expressed in this article are solely those of the authors and do not necessarily represent those of their affiliated organizations, or those of the publisher, the editors and the reviewers. Any product that may be evaluated in this article, or claim that may be made by its manufacturer, is not guaranteed or endorsed by the publisher.
