# Supplementary material for: Cardiac phase-resolved late gadolinium enhancement imaging
Source: Front Cardiovasc Med. 2022 Sep 29;9:917180. doi: 10.3389/fcvm.2022.917180 (PMC9557076; doi:10.3389/fcvm.2022.917180)
Supplement: Supplementary file 1 [file Data_Sheet_1.pdf]

Supplementary Material

1 SUPPLEMENTARY DATA

1.1 Figures

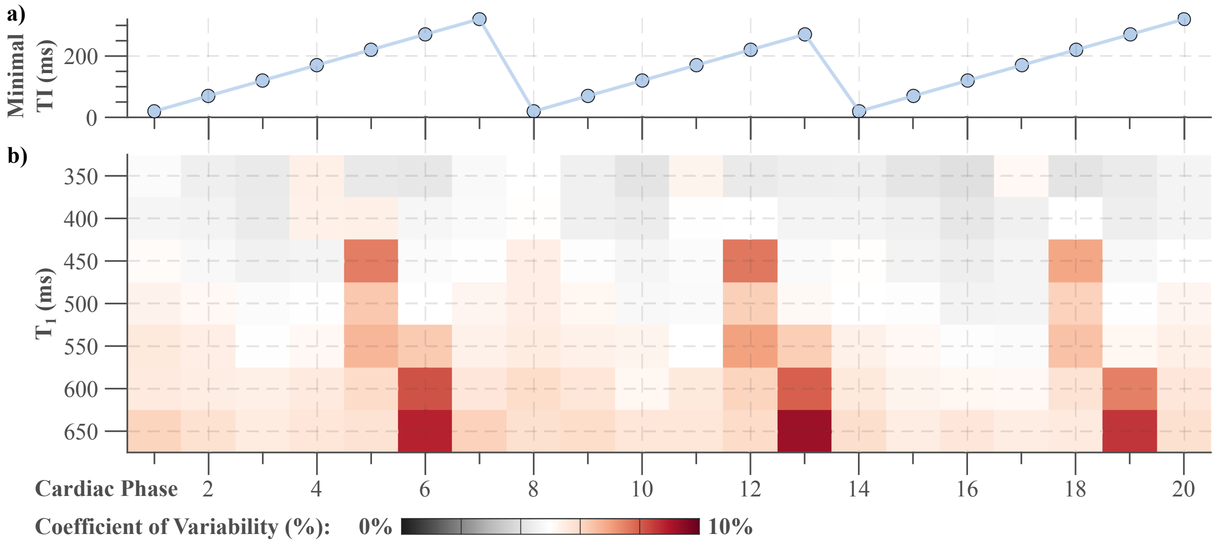

Figure S1. a) Minimal inversion time (TI), characterizing the sampling pattern of the inversion recovery curve for each cardiac phase. Different sampling schemes are realized for different cardiac phases. As three inversion pulse offsets are played, a three-times repeating pattern is obtained for the minimal TI. b) Coefficient of variability describing the noise resilience in a simulated functional LGE acquisition. Large differences in noise susceptibility are observed among the cardiac phases. The highest noise variability is observed for cardiac phases, where the minimal TI is close to the nulling point of the simulated T_1 time. Additionally, longer simulated T_1 times, lead to an overall increase in noise variability.

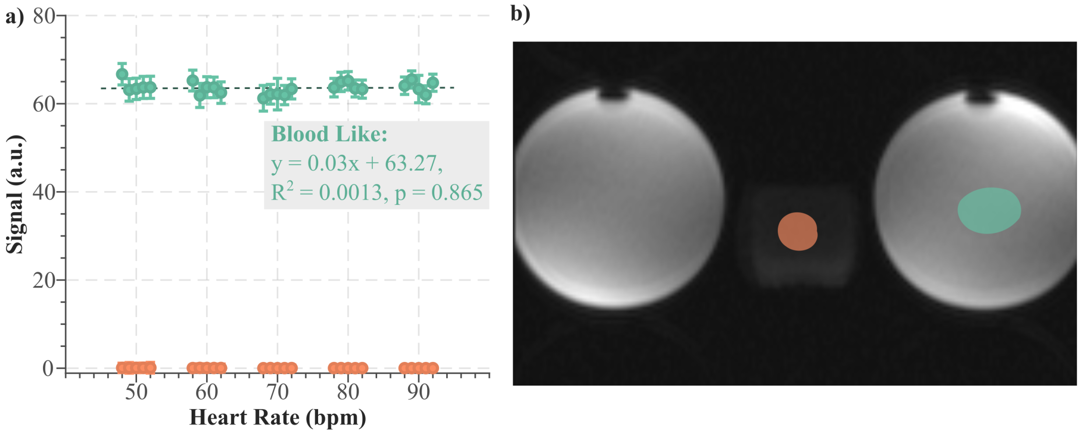

Figure S2. a) Signal of the functional LGE images acquired in phantom at various simulated heart rates. Five repetitions were performed for each heart rate, and the contrast was synthesized to null the myocardium-like compartment (orange ROI). The signal in the blood-like compartment (green ROI), shows no significant trend across different heart rates. b) Example baseline image of the phantom, highlighting the ROIs in the myocardium and blood-like compartments.

1.2 Videos

Supplementary Video S1 Cardiac phase-resolved LGE images in a cinematographic view, acquired with the proposed technique in three healthy subjects at 3T with a temporal resolution of 40 ms.

Supplementary Video S2 Phase-resolved LGE images acquired at 1.5T with a temporal resolution of 80 ms in two patients that depicted no scar.

Supplementary Video S3 Phase-resolved LGE images acquired in two scar patients at 1.5T.
